# Trends in the China pathologist workforce From 2010 to 2022

**DOI:** 10.3389/frhs.2026.1812918

**Published:** 2026-06-10

**Authors:** Zhun Shu, Zebiao Liu, Jianzhu Zhang, Wenyan Sui

**Affiliations:** Department of Pathology, Huizhou First Hospital, Huizhou, Guangdong, China

**Keywords:** China, pathologist, tumor patient, workforce, workload

## Abstract

**Background:**

Pathology faces a global personnel shortage and increasing workload due to rising cancer cases and diagnostic complexity, yet data in China are lacking. This study examines trends in China's pathology workforce from 2010 to 2022.

**Methods:**

Data during 2010-2022 were collected from the China Health Statistical Yearbook, including the proportion of pathologists among physicians, oncology clinic outpatients, and discharged tumor patients. Data on new cancer cases and incidence rates from 2010 to 2018 were obtained from the China Cancer Registry Annual Report. We calculated ratio metrics (workload per pathologist) and growth rates, and performed descriptive linear regression to assess workforce demand balance.

**Results:**

From 2010 to 2022, the proportion of pathologists among physicians remained stable in China, while the estimated absolute number increased. Concurrently, the estimated national new cancer cases rose. Due to the increase in pathologist density (per million population), the estimated average cancer case load per pathologist decreased from 331.86 in 2010 to 215.29 in 2018. However, from 2010 to 2022, oncology clinic outpatients and inpatient tumor patients per pathologist increased by 131.42% and 46.98%, respectively. Growth rate analyses showed that oncology clinic outpatients and inpatient tumor patients increased by 325.28% and 170.10%, respectively, whereas the pathologist workforce increased by 83.77%. Descriptive linear regressions indicated positive correlations between pathologist numbers and patient volumes (all *P* < 0.001).

**Conclusions:**

Although the pathologist workforce has grown in China, its growth has not kept pace with the rising diagnostic demand. The correlation between tumor patient volume and pathologist numbers highlights the need for workforce planning and efficiency-enhancing technologies.

## Introduction

Pathology is the cornerstone of cancer diagnosis (1), as its accuracy correlates with appropriate treatment and influences patient prognosis. However, many countries have experienced shortages of pathologists in recent years. This shortage reflects not a decline in numbers, but the growing in the demand and complexity of diagnostic services, resulting in increased workload of pathologists.

Moreover, this burden is a common challenge faced by pathology services worldwide. In 2017, there were only 3.94 pathologists per 100,000 people in America, lower than Canada (4.81 per 100,000 people) (2). Although the number of pathologists increased to 21,215 in America in 2022 according to Association of American Medical Colleges (AAMC) data, their population density still remains low at 6.4 per 100,000 people (3). Similarly, there is only one pathologist for every 47,989 people in Germany (4). In addition, while the number of pathologists increased by nearly 13% in Canada during 1999–2009, newly diagnosed cancer cases rose by over 32%, leading to a 17.1% increase in workload per pathologist (5). Metter et al., found that there was a positive correlation between the rise in new cancer cases and pathologist workload, showing that less than 29% increase in new cancer cases led to a 7.06% rise in workload per pathologist (3).

In addition to the absolute increase in tumor cases, the workload burden is also intensified by the increasing complexity of diagnoses. Over the past two decades, pathology has transformed from descriptive H&E-based diagnosis into a complex field, utilizing diverse tools to generate diagnostic, predictive, prognostic, and therapeutic biomarkers (6). While this advancement provides more comprehensive diagnostic information, it has also increased case complexity and pathologist workload. A study reported that during 2006–2014 the number of slides increased by over 60%, immunohistochemical slides nearly doubled, and molecular tests more than tripled at an institution in Germany (7).

Unlike clinical departments, the workload of pathologist is often underrecognized and relevant to the volume of cases of clinical departments. However, data on pathologist workload in China is lacking. Therefore, this study aims to provide evidence for staffing in pathology departments, improving diagnostic efficiency, and introducing innovative solutions such as artificial intelligence (AI) by analyzing data on pathologists and cancer patients in China.

## Material and methods

### Data source

Data were obtained from the China Health Statistics Yearbook (CSY) (https://www.nhc.gov.cn/mohwsbwstjxxzx/tjzxtjsj/tjsj_list.shtml), CSY, compiled by the National Health Commission of the People's Republic of China, is a national annual survey report reflecting the development of healthcare systems and the current health status of residents in 31 provinces, autonomous regions and municipalities in Mainland China. The database contains the number of assistant physicians, licensed physicians, the proportion of pathologists, and outpatient visits to oncology departments, as well as discharged tumor patients from public hospitals. The annual data from 2010 to 2022 from the CSY were used to analyze the trends of the pathologist workforce and tumor patients over the past 13 years. In China, licensed pathologists hold a bachelor's degree or higher and practice independently, while assistant pathologists typically hold a junior college degree and practice under supervision, except in rural regions. Pathologists were not directly reported, we estimated the annual number of pathologists as: *Pathologists* *=* *Total physicians×Percentage of pathologists among physicians.* China Cancer Registry Annual Report, compiled by National Cancer Center, includes the population coverage, number of new cancer cases, and the national cancer incidence rate. The most recent data covering the period up to 2018 are publicly and freely accessible. No ethical permission is required for the publicly downloaded data.

### Statistical analyses

All statistical analyses and data visualization were performed using Microsoft Excel 2019 (Microsoft Corp., USA) and Origin 2021 (OriginLab Corp., USA), respectively.

## Results

### Pathologist workforce profile

**Figure 1:**
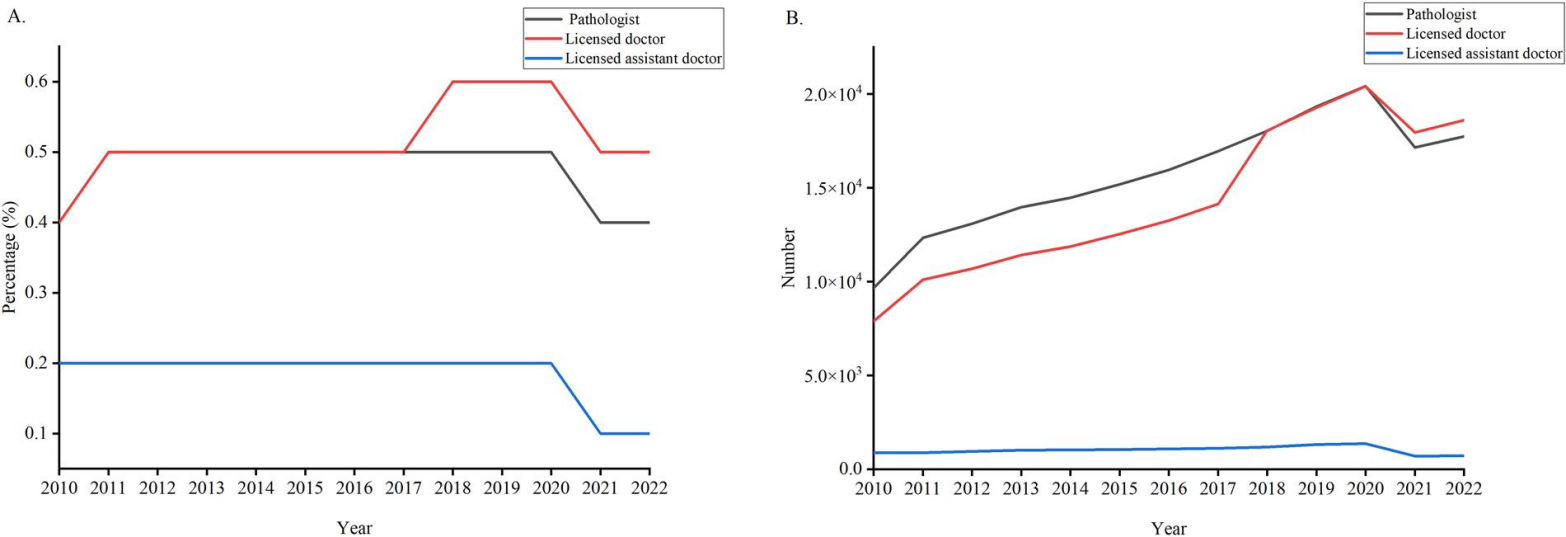
During 2010 to 2022, pathologists maintained a stable proportion of physicians **(A)**, the number of pathologists is gradually increasing **(B)**.

During 2010-2022, the physician workforce has grown dramatically in China. At the end of 2022, the number of physicians reached over 4 million, which is 1.8 times more than that in 2010. Among them, the number of licensed physicians was over 3.7 million, also 1.8 times that of 2010. Over this period, pathologists (licensed and assistant) maintained a stable share of the total physician workforce (Figure 1A). Thus, their absolute number, calculated by this stable proportion, continued to rise as a result of the growing overall physician workforce (Figure 1B).

### Cancer incidence and pathologist density

**Figure 2:**
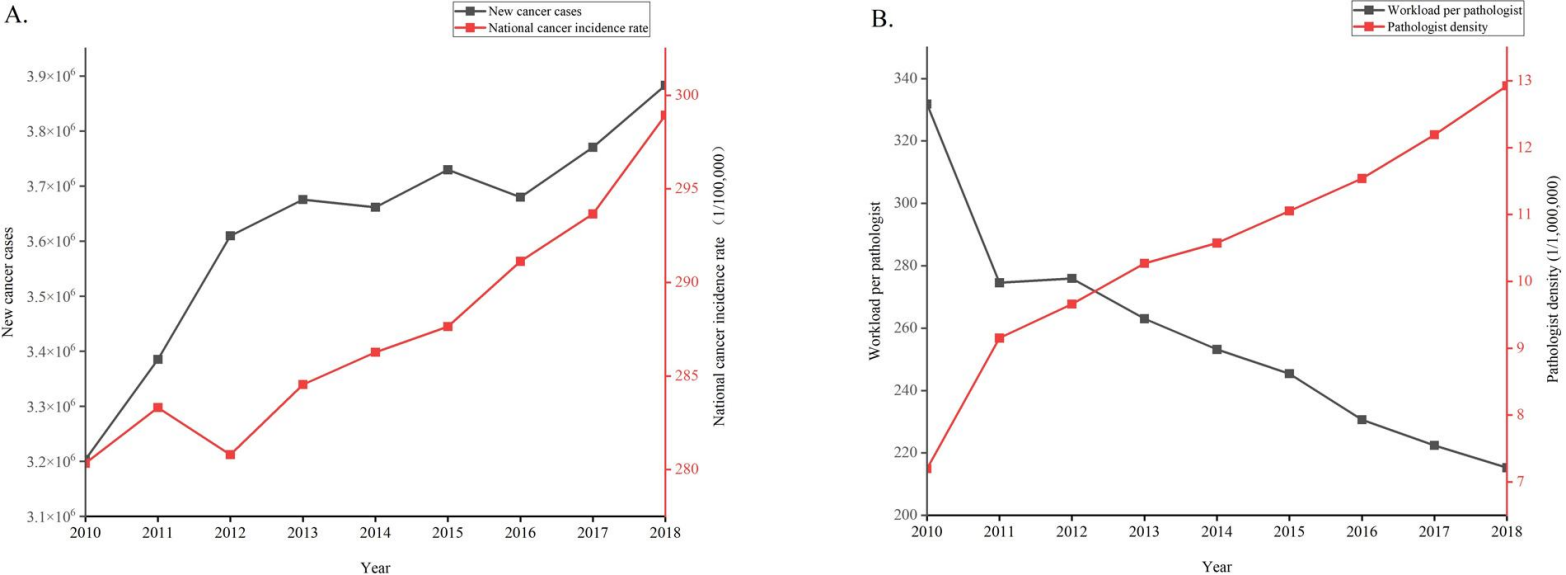
During 2010 to 2018, trends in cancer incidence and estimated new cases based on data from China cancer registry annual report **(A)**, workload per pathologist and pathologist density **(B)**.

**Figure 3:**
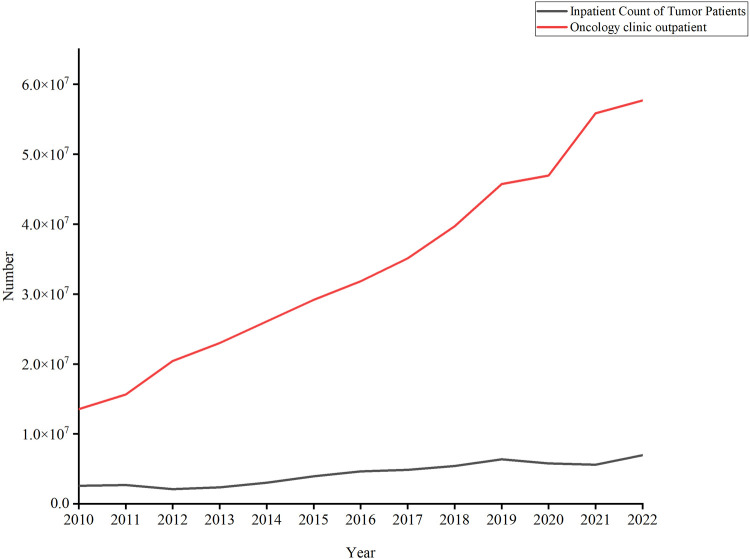
During 2010 to 2022, growth in oncology clinic outpatients and public hospitals cancer discharges.

According to the China Cancer Registry Annual Report, the annual number of newly reported cancer cases rose from 495,069 in 2010 to 1,765,264 in 2018, with the incidence rate increasing from 280.34 to 298.94 per million population. Based on this data, the estimated national number of new cancer cases increased from approximately 3.2 million (3,203,421) in 2010 to approximately 3.88 million (3,882,890) in 2018 (Figure 2A). Concurrently, the pathologist workforce expanded, with their density increased from 7.20 to 12.93 per million population. Consequently, the estimated workload per pathologist for new cancer cases decreased from 331.86 to 215.29 during 2010-2018 (Figure 2B). Parallel to these trends, oncology clinic outpatients increased from 13,561,601 to 57,674,367 during 2010-2022, while cancer discharges from public hospitals rose from 2,584,188 to 6,979,931(Figure 3).

### Pathologist workforce and tumor patient volume

**Figure 4:**
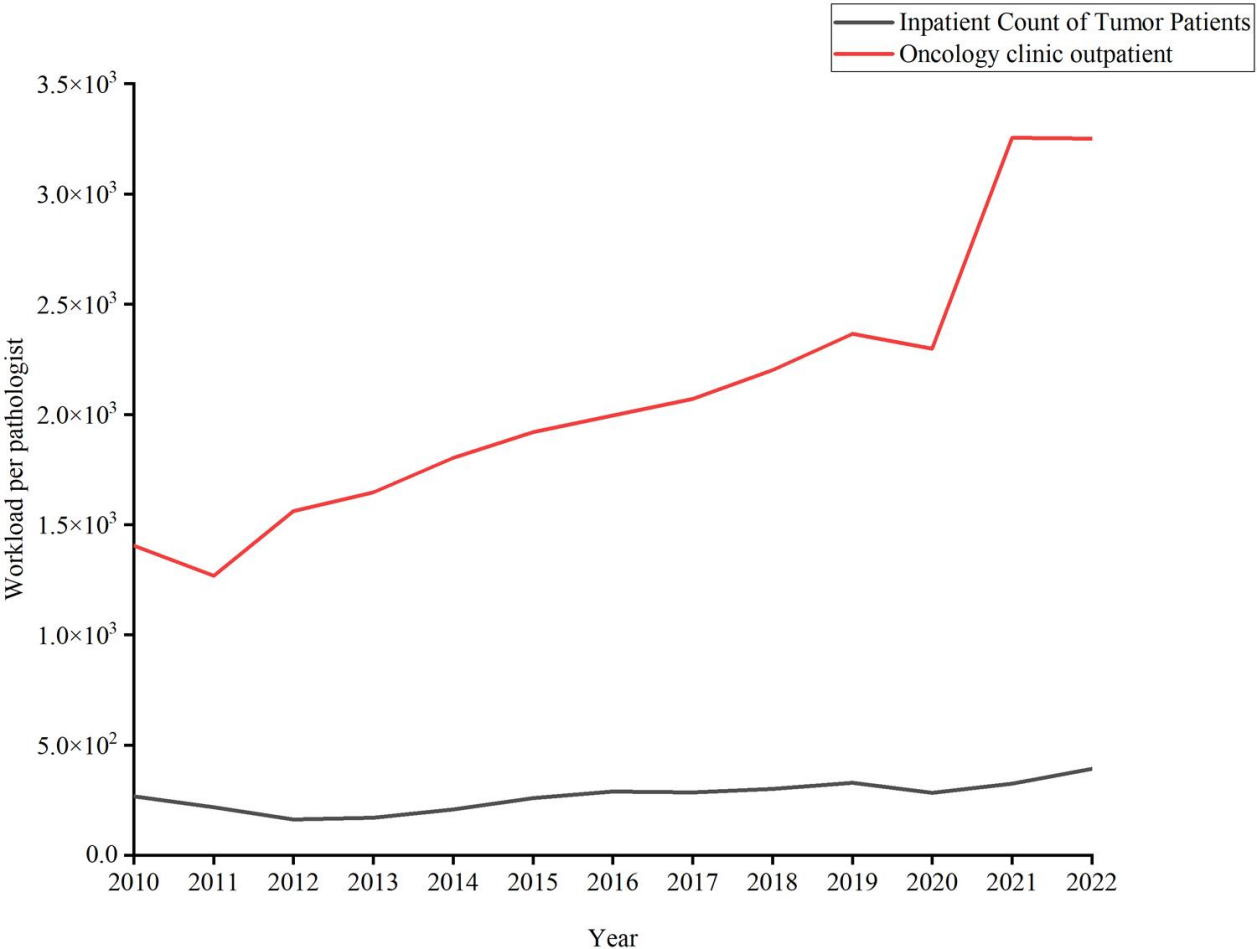
During 2010 to 2022, trends in workload per pathologist (oncology clinic outpatients and inpatient tumor patients) in China.

To assess the relationship between pathologist workforce and tumor diagnostic demand, we examined ratio metrics across different indicators. Ratio metrics showed that between 2010 and 2022, oncology clinic outpatients per pathologist increased from 1,404.91 to 3,251.29 (a 131.42% increase), and inpatient tumor patients per pathologist increased from 267.71 to 393.48 (a 46.98% increase) (Figure 4). And for new cancer cases (2010–2018), as shown in Figure 2B, workload per pathologist declined.

**Figure 5:**
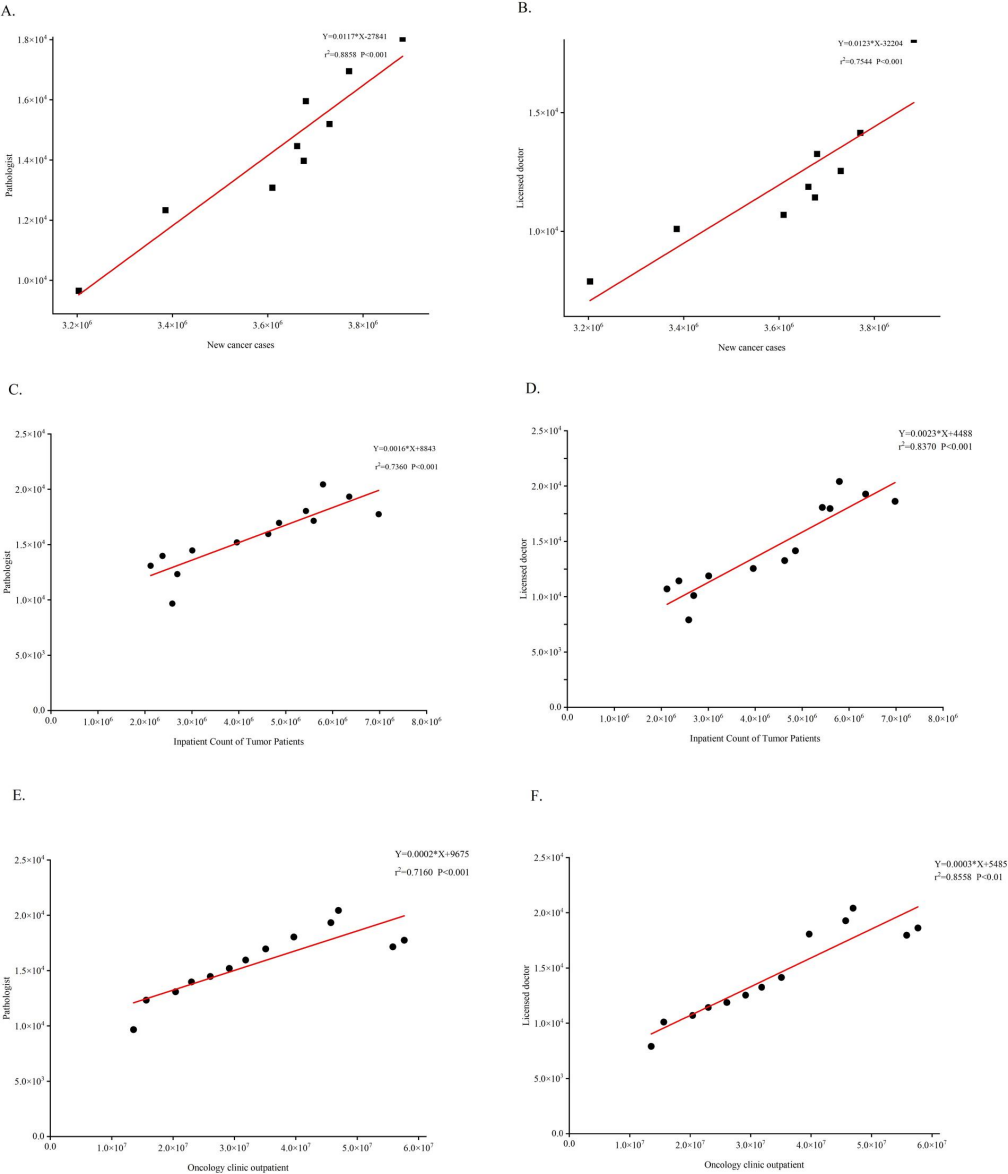
Total pathologists and licensed pathologists numbers show significant correlation with new cancer cases **(A,B)**, inpatient and outpatient tumor patients **(C–F)**.

As a descriptive observation, the estimated numbers of total pathologists and licensed pathologists both showed a statistically significant proportional relationship with the estimated national numbers of new cancer cases (*β* = 0.0117, 95% CI: 0.0082–0.0151, r^2^ = 0.8858 and *β* = 0.0123, 95% CI: 0.0065–0.0180, r^2^ = 0.7544, both *P* < 0.001) (Figures 5A,B). Similarly, their inpatient and outpatient tumor patients (for pathologists: *β* = 0.0016, 95% CI: 0.0010–0.0022, r^2^ = 0.7360 and *β* = 0.0002, 95% CI: 0.0001–0.0002, r^2^ = 0.7160; for licensed pathologists: *β* = 0.0023, 95% CI: 0.0016–0.0029, r^2^ = 0.8370 and *β* = 0.0003, 95% CI: 0.0002–0.0003, r^2^ = 0.8558; all *P* < 0.001) (Figures 5C–F).

However, because regression of two time-trending variables may produce spurious correlations, we also performed growth rate analyses. Cumulatively, oncology clinic outpatients increased by 325.28% over the study period, whereas the pathologist workforce increased by 83.77%. Similarly, inpatient tumor patients grew by 170.10%, far exceeding workforce growth rate of 83.77%. Overall, the rising per-capita workload and persistently higher demand growth rates indicate that the expansion of the pathologist workforce has not fully kept pace with the growing diagnostic demand.

## Discussion

There was an imbalance between the growths in the number of pathologists and demand for diagnostic services. Our study shows that from 2010 to 2022, the total number of licensed (including assistant) pathologists in China increased from an estimated 9,653 to approximately 17,739, an absolute increase of about 8,100 pathologists. However, this growth did not keep pace with the concurrent rise in cancer cases. Based on data from the China Cancer Registry Annual Report, the annual number of cancer patients rose by approximately 680,000 (from 3.2 million to 3.9 million) between 2010 and 2018. This means that each additional pathologist faced roughly 84 new cancer patients annually, reflecting persistent diagnostic strain. Furthermore, the scale of demand is underscored by the 2020 GLOBOCAN estimates, which reported approximately 4.82 million new cancer cases in China in 2022 (8). Furthermore, our linear regression analysis revealed a statistically significant proportional relationship between the number of pathologists and the national estimates of new cancer cases, inpatients, and outpatients with tumors. These results indicate that the tumor patient load is an important factor for the distribution of pathology human resources. As a descriptive observation, the results of linear regression showed positive correlations between pathologist numbers and patient volumes, but such correlations do not imply causation due to shared time trends. Instead, ratio metrics and growth rate analyses indicated that demand growth outpaced workforce expansion. Notably, although the estimated workload per pathologist for new cancer cases decreased from 331.86 to 215.29 during 2010-2018, this simplistic metric fails to capture the actual workload. While the workload of pathologists merely reflected with the number of patients seen, in reality, it is embodied in each clinical specimen and slide, as well as the required effort and complexity of every individual case. Besides the shortage of pathologists, standard morphological descriptions of cancer specimens are becoming increasingly complex, with a growing annual volume of ancillary diagnostics, immunohistochemistry, and molecular detection. These lead to a higher workload and greater complexity for pathologists (9). In the context of rising diagnostic demands, pathology faces a global workforce crisis. For instance, the number of pathologists is projected to decline from 5.7 to 3.7 per 100,000 population between 2010 and 2030 in the United States (9). Consequently, despite the continuous expansion of the pathologist workforce, the density of pathologists per million population remained comparatively low in our study, ranging from 7.2 to 12.92. Since case complexity was not directly measured in this study, the actual workload of pathologists in China may be underestimated.

With an increasing workload, a range of negative outcomes will appear, such as overwork exposes physicians to serious health risks, which may lead to lower quality services for patients (10). A study indicates that doctors who work for more than 45 h a week are more likely to commit medical errors than those who work for less than 45 h a week (11). Furthermore, long working hours and heavy workload were considered as risk of death from overworked, 207 abnormal deaths of physicians had been reported in mainland China from 2007 to 2020, and most deaths (∼79%) were due to overwork or sudden death (10). These alarming findings are relevant to pathology, one of the specialties hardest hit by workforce shortages and workload pressures, underscoring the urgent need to address workload pressures in pathology.

Traditional histology and pathology have played a key role in cancer diagnosis and classification for decades (12). As the shortage of pathologists and the population ages, burden of cancer diagnosis keeps growing, digital pathology and artificial intelligence technology have emerged, which can reduce the work burden of pathologists and improve diagnostic accuracy (12). Digital pathology transmits digital slides directly to pathologists in a paperless manner, which can reduce the risk of tissue/slide loss or damage, enable rapid case tracking, archiving, and retrieval, improve diagnostic efficiency, and provide a more flexible working mode for pathologists (9). Digital pathology can reduce turnaround time for diagnosis. For example, in consecutive consultations of head and neck pathology, digital pathology reduced the median turnaround time by 97% (13). Apart from that, AI further enhanced digital pathology power, which has been considered the third revolution of pathology (14). AI can improve diagnostic efficiency by automating various tasks (15), which decreased time to identify gastric cancer lesions by 99.43% and diagnose in prostate biopsy by 21.94% (16). The application of AI will be a critical solution for alleviating the workload pressure on pathologists in China and coping with the increasing workload.

In this study, we have quantified the increasing workload burden of pathologists in China by analyzing data from China's healthcare system from 2010 to 2022. We found that the pathologist workforce may not have kept pace with the growing diagnostic demand, leading to increased workload per capita. This could potentially affect the quality and sustainability of China's cancer care system. Such risks include a higher rate of diagnostic error, delayed reports, and physician burnout, all of which could compromise patient outcomes. To address this challenge, a multi-faceted strategy is required. Firstly, expand and stabilize the pathologist workforce through medical education and career incentives. Secondly, optimize workflows to improve diagnostic efficiency. Finally, promote technological innovation, particularly the development and clinical application of AI-assisted diagnostic tools.

Although this study has quantified the increasing workload burden of pathologists, the findings are subject to several limitations. Firstly, the number of pathologists was estimated based on the total physician count and the proportion of pathologists. And sensitivity analysis is not feasible because national pathologist counts are not available directly. Secondly, due to the unavailability of national data on biopsies, immunohistochemistry, molecular tests, frozen sections, consultations, teaching, and administration, we used new cancer cases, oncology clinic outpatients and inpatient tumor patients per pathologist as workload metrics, which likely underestimates the true burden on pathologists. Thirdly, due to data unavailability, we did not analyze variations in workload by region, hospital level, or subspecialty. National averages may conceal important geographic variation in pathology workforce shortages. Fourthly, we could not perform multivariable adjustment for population growth, aging, healthcare expansion, or policy shifts due to a lack of consistent annual data aligned with our workforce dataset. Finally, we did not validate occupational coding; thus, workforce counts may include non-diagnostic staff or miss some pathologists.

## Data Availability

The original contributions presented in the study are included in the article/Supplementary Material, further inquiries can be directed to the corresponding author.
